# Efficacy and safety of non-invasive neurostimulation for insomnia in adults: protocol of a systematic review and network meta-analysis

**DOI:** 10.3389/fpsyt.2025.1691844

**Published:** 2025-11-26

**Authors:** Jinxian Lu, Jun Hu, Yi Lu, Xuecheng Zhang, Qi Wang, Hongling Jia, Yongchen Zhang

**Affiliations:** 1 Department of Acupuncture, The Second Affiliated Hospital of Shandong University of Traditional Chinese Medicine, Jinan, Shandong, China; 2 School of Acupuncture-Moxibustion and Tuina, Shandong University of Traditional Chinese Medicine, Jinan, Shandong, China; 3 Department of Radiology, The Second Affiliated Hospital of Shandong University of Traditional Chinese Medicine, Jinan, Shandong, China; 4 Department of Physical Therapy, Shandong Mental Health Center, Shandong University, Jinan, Shandong, China; 5 Department of Proctology, China-Japan Friendship Hospital, Beijing, China; 6 Second School of Clinical Medicine, Shandong University of Traditional Chinese Medicine, Jinan, Shandong, China; 7 Department of Acupuncture, Affiliated Hospital of Shandong University of Traditional Chinese Medicine, Jinan, Shandong, China

**Keywords:** insomnia, non-invasive neurostimulation, brain stimulation, sham intervention, systematic review

## Abstract

**Background:**

Non-invasive neurostimulation therapies have demonstrated broad therapeutic potential in the management of insomnia. However, there remains a paucity of systematically integrated evidence regarding the efficacy of commonly used clinical interventions. To address this gap, this study will employ systematic review and network meta-analysis (NMA) to evaluate the comparative efficacy and safety profiles of different non-invasive neurostimulation for insomnia, with the aim of providing evidence-based guidance for clinical decision-making.

**Methods:**

The PubMed/Medline, Web of Science, Embase, CENTRAL, Scopus, and PsycINFO databases will be systematically searched for randomized controlled trials (RCTs) on non-invasive neurostimulation techniques for the treatment of insomnia in adults, published from inception to August 31, 2025, with no language restrictions. The primary outcome measure is the Pittsburgh Sleep Quality Index (PSQI) score. Secondary outcomes include the Insomnia Severity Index (ISI) score, emotional symptoms, quality of life, and incidence of adverse events. Two independent researchers will perform literature screening, data extraction, and risk of bias assessment. The overall quality of evidence will be evaluated using the CINeMA. For NMA, we will use a random-effects model based on the Bayesian approach to integrate direct and indirect evidence. Statistical analysis will be performed using R software, and the consistency and heterogeneity of the model will be tested.

**Discussion:**

This study will provide an up-to-date synthesis of evidence from all relevant RCTs, which is crucial for evaluating the therapeutic potential and adverse effects of non-invasive neurostimulation techniques in insomnia treatment. The findings will offer valuable insights to assist clinicians in optimizing evidence-based practice.

**Systematic Review Registration:**

https://www.crd.york.ac.uk/prospero/, identifier CRD420251084949.

## 1 Introduction

Insomnia is a prevalent clinical condition characterized by difficulty initiating or maintaining sleep, accompanied by symptoms such as daytime irritability and fatigue. This disorder may present as an independent condition or coexist with other medical and mental health comorbidities, representing a significant risk factor for psychological impairment and functional disability. Epidemiological studies estimate its prevalence at 10%-20% in the general population, with approximately 50% of cases following a chronic course. Without proper intervention, insomnia can substantially impair patients’ quality of life (1, 2).

Modern medicine primarily employs cognitive behavioral therapy (CBT) and oral medication as main interventions. Although it can achieve certain results, CBT reduces patient acceptance and compliance due to time-consuming travel costs and a shortage of professionals, and long-term medication also produces adverse events such as next-day residual effects, withdrawal symptoms, physiological tolerance, and side effects (3, 4); therefore, seeking safe and effective alternative therapies or adjunctive therapy has become an important issue in the management of insomnia.

As an emerging intervention in sleep medicine, non-invasive neurostimulation improves sleep parameters by targeting the excitability of the cerebral cortex, mainly including brain stimulation (e.g., transcranial magnetic stimulation, transcranial electrical stimulation) and nerve stimulation (e.g., transcutaneous auricular vagus nerve stimulation, vestibular nerve stimulation), the safety and feasibility of which have been supported by related studies (5, 6). Despite these encouraging findings, the clinical translation of non-invasive neurostimulation faces several challenges. Methodological heterogeneity across studies—including variations in stimulation parameters, sample sizes, blinding procedures, and control for placebo effects—has contributed to inconsistent findings and generally limited the quality of evidence (7). Furthermore, although the number of clinical studies has grown substantially in recent years, existing systematic evaluations and meta-analyses (8–11) have been restricted to assessing individual intervention modalities, lacking comprehensive comparisons across different neurostimulation approaches. This evidence gap significantly hinders evidence-based treatment selection and the refinement of protocols. It is worth noting that the field continues to evolve, with emerging technologies such as temporal interference (TI) stimulation and transcranial focused ultrasound (tFUS) beginning to demonstrate potential for sleep regulation (12, 13). However, randomized controlled trials applying these novel techniques specifically to insomnia remain limited; thus, they fall beyond the scope of the current protocol while representing an important direction for future research (14, 15).

To address these evidence gaps, this study will employ NMA to integrate the latest research data, enabling for the first time both direct and indirect comparisons among common non-invasive neurostimulation techniques. Through comprehensive evaluation of their efficacy and safety profiles, we aim to provide evidence-based guidance for clinical decision-making. Furthermore, our findings may facilitate protocol optimization to improve long-term prognosis and quality of life in patients with insomnia.

## 2 Methods

The protocol adheres to the Preferred Reporting Items for Systematic Review and Meta-Analysis Protocols (PRISMA-P) guidelines for systematic review protocols (16) (checklist provided in **Supplementary Appendix 1**), while the subsequent NMA will follow the PRISMA-NMA extension (17). The study protocol has been prospectively registered on the PROSPERO platform (registration number: CRD420251084949).

### 2.1 Inclusion and exclusion criteria

According to the PICOS (Population-Intervention-Comparison-Outcomes-Study design) principle, the inclusion criteria will be designed in the following five components:

#### 2.1.1 Population

According to the Diagnostic and Statistical Manual of Mental Disorders (DSM) (18–20), the International Classification of Sleep Disorders (ICSD) (21, 22), or other recognized diagnostic criteria, patients diagnosed with primary insomnia or chronic insomnia disorder as the main condition (≥ 18 years old) were included, regardless of gender, race, economic status, or the severity of insomnia. We will exclude studies focusing on participants with insomnia secondary to other medical or psychiatric conditions (where insomnia is not the primary concern), subclinical insomnia, or other primary sleep disorders (e.g., narcolepsy, sleep apnea). However, to enhance the clinical representativeness of our findings, studies including participants with stable and mild-to-moderate comorbid anxiety or depressive symptoms (as defined in the original studies, e.g., below clinical cutoff on standardized scales) will be included, provided that insomnia disorder remains the primary diagnosis requiring intervention.

#### 2.1.2 Intervention/comparison

Through a systematic preliminary search, we identified eligible neurostimulation techniques, including non-invasive brain and nerve stimulation approaches, as falling within the scope of our study. The specific measures for the intervention group are as follows:

1. Transcranial magnetic stimulation (TMS): TMS involves applying electromagnetic pulses through a coil to target specific brain regions. Different protocols are formed based on variations in pulse frequency and pattern (e.g., low-frequency or high-frequency repetitive TMS [rTMS] and theta burst stimulation (TBS), which delivers TMS pulses at gamma frequencies (e.g., 50 Hz) with repetitions at theta frequencies (e.g., 5 Hz) (23, 24).
2. Transcranial Electrical Stimulation (tES): tES applies weak currents through bipolar electrodes on the scalp to stimulate specific brain regions. It primarily includes two forms: transcranial direct current stimulation (tDCS) and transcranial alternating current stimulation (tACS) (25, 26).
3. Transcutaneous auricular vagus nerve stimulation (taVNS): Electrodes are placed in the patient’s external auditory canal to apply electrical stimulation to the auricular branch of the vagus nerve at different frequencies and intensities, inducing central or peripheral effects (27–29).
4. Vestibular nerve electrical stimulation (VeNS): Electrodes are placed behind the ear on the mastoid process to stimulate branches of the vestibular nerve. By controlling the intensity level of stimulation, brain functional activity can be regulated (30, 31).

Intervention studies will exclude those combining drugs, psychotherapy, or other therapies, as well as those comparing different treatment durations or frequencies. Control groups will primarily use sham stimulation or placebo controls. Studies using no treatment, waiting lists, or routine care as controls will be excluded.

#### 2.1.3 Primary outcome

The primary outcome will be the change in sleep quality as measured by the Pittsburgh Sleep Quality Index (PSQI) from baseline to post-intervention. The PSQI was selected as it provides a comprehensive, multi-dimensional assessment of sleep quality over the past month, encompassing key domains such as sleep latency, duration, efficiency, and daytime dysfunction. A change in the PSQI score is clinically interpretable and reflects an overall improvement in the patient’s sleep experience and daytime functioning (32). For the primary analysis, the post-intervention time point of interest is defined as the assessment conducted closest to the end of the intervention course, within a window of immediately after the final session to 1 month post-treatment.

#### 2.1.4 Secondary outcomes

1. The use of the ISI to assess the reduction in insomnia symptoms before and after the intervention (33);
2. The impact on emotional symptoms: measuring the impact of emotional changes in patients through standardized questionnaires such as the Hamilton Depression Rating Scale (HAMD) (34) or the Hamilton Anxiety Rating Scale (HAMA) (35);
3. Health-related quality of life (HRQoL): assessed using any validated global tools, such as the 36-Item Short Form Health Survey (SF-36) (36) or the WHO quality of life questionnaire (WHOQOL) (37).

Additionally, longer-term follow-up data for these outcomes (e.g., >1 month post-intervention) will be extracted and analyzed separately to explore the sustainability of effects.

#### 2.1.5 Safety outcomes

1. Tolerability: The proportion of participants experiencing at least one treatment-related adverse event (AE);
2. Serious harms: The proportion of participants experiencing at least one serious adverse event (SAE), analyzed separately. SAEs will be defined according to the International Conference on Harmonisation (ICH) guidelines or the definitions provided in the original studies (38);
3. Descriptive safety profile: The nature (type), frequency, and severity of specific adverse events (e.g., headache, skin irritation) will be systematically recorded for descriptive summary by intervention.

#### 2.1.6 Study design

We will include RCTs using either parallel or crossover designs. For crossover trials, only data from the first period will be analyzed to avoid potential carryover effects (39). Non-RCT studies, quasi-randomized controlled trials, case reports, reviews, conference abstracts, editorials, animal studies, mechanistic studies, duplicate publications, and studies with missing outcome measures or incomplete data will be excluded.

### 2.2 Information sources and search strategy

We will conduct a comprehensive literature search across six electronic databases from inception to August 31, 2025: PubMed/Medline, Web of Science, Embase, CENTRAL, Scopus, and PsycINFO. Additionally, we will systematically search trial registries (ClinicalTrials.gov and the WHO International Clinical Trials Registry Platform [ICTRP]) to identify both ongoing and completed but unpublished trials. For completed trials that meet our eligibility criteria but lack published results in academic journals, we will implement the following procedure to minimize publication bias:

1. We will first download any available results summary directly from the registry entry.
2. If no results are available in the registry, we will contact the principal investigators or sponsors via email to request the necessary data for inclusion in our meta-analysis.
3. Unpublished trials for which sufficient outcome data can be obtained will be included in the quantitative synthesis. We will transparently report the process and outcomes of this data acquisition effort in the final review.

We will also manually screen the reference lists of previous systematic reviews to locate potentially relevant studies. There will be no restrictions on language, and the search strategy for the PubMed database is illustrated in **Table 1**.

**Table 1:** Search strategy used in PubMed database.

Number	Search terms
#1	Sleep Initiation and Maintenance Disorders [MeSH]
#2	insomnia [Title/Abstract]
#3	sleep disorder* [Title/Abstract]
#4	sleep disturb* [Title/Abstract]
#5	sleepless* [Title/Abstract]
#6	#1 OR #2 OR #3 OR #4 OR #5
#7	(non-invasive brain stimulation OR non-invasive nerve stimulation
	OR non-invasive neuromodulation OR NIBS) [Title/Abstract]
#8	(transcranial magnetic stimulation OR theta burst stimulation OR TMS OR
	TBS) [Title/Abstract]
#9	(transcranial direct current stimulation OR transcranial alternating current
	stimulation OR tDCS OR tACS) [Title/Abstract]
#10	(transcutaneous auricular vagus nerve stimulation OR vagus nerve stimulation
	OR taVNS OR VNS) [Title/Abstract]
#11	(vestibular nerve stimulation OR galvanic vestibular stimulation OR VeNS OR GNS) [Title/Abstract]
#12	#7 OR #8 OR #9 OR #10 OR #11
#13	Randomized controlled trial [Publication Type]
#14	random* [Title/Abstract]
#15	placebo [Title/Abstract]
#16	double-blind [Title/Abstract]
#17	#13 OR #14 OR #15 OR #16
#18	#6 AND #12 AND #17

### 2.3 Study selection and data extraction

**Figure 1:**
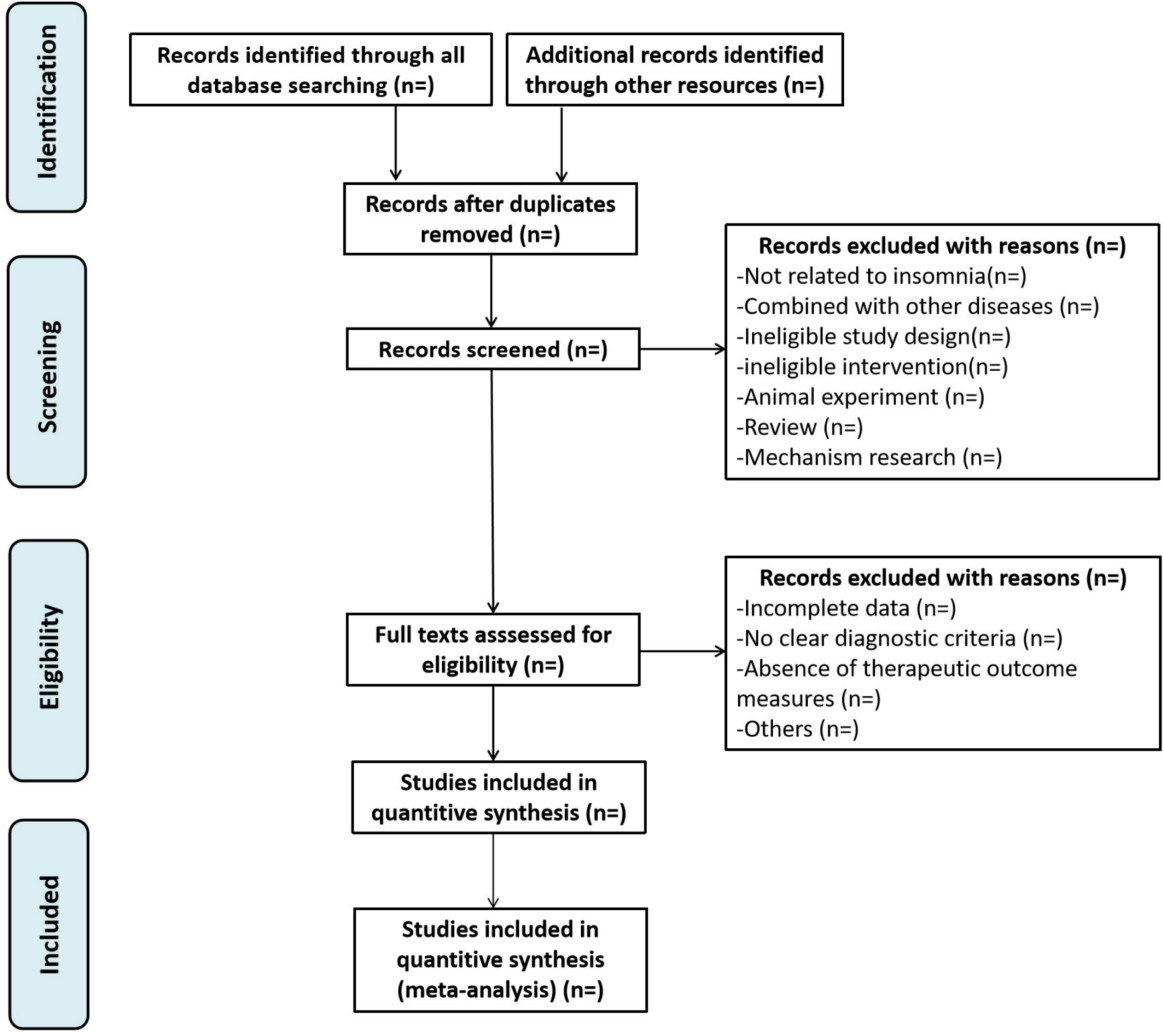
The PRISMA flow diagram.

According to the above search strategy, the downloaded literature records will be imported into EndNote X9 software for unified management and duplicate studies will be removed. Two researchers (JXL and XCZ) will independently screen the literature that meets the requirements. For uncertain or disputed studies, consensus will be reached through joint discussion or consultation with a third party (HLJ). The entire process will strictly follow PRISMA guidelines, as shown in **Figure 1**.

Data extraction will be performed using standardized tables in Microsoft Excel, with the process conducted independently by two researchers (JXL and XCZ) and cross-checked to ensure the completeness and accuracy of the information. Extracted content will include basic study characteristics (first author, publication year, country, study type, number of trial arms, insomnia type, and diagnostic criteria) as well as information related to the treatment group and control group (age, sample size, treatment duration, adverse event reporting, and outcome measures). Any questions will be resolved through negotiation by a third party (HLJ). For studies that are otherwise eligible but have missing or incomplete outcome data (e.g., standard deviations, correlation coefficients for change scores), we will initially contact the corresponding authors via email to request the necessary information. If no response is received after two reminders (spanning 4 weeks), we will implement the following pre-specified imputation strategy, as recommended by the Cochrane Handbook (40): Standard deviations will be estimated from confidence intervals (CIs), standard errors (SEs), or p-values when available; if these statistics are not reported, we will borrow the average standard deviation (SD) from other comparable studies in our review that used the same outcome instrument and featured a similar study population in terms of baseline severity and key characteristics. The impact of any imputation on the primary results will be assessed in a sensitivity analysis.

### 2.4 Methodological quality assessment

Two researchers (JXL and XCZ) will independently assess the quality of included studies using the Cochrane Collaboration’s risk of bias assessment tool 2.0 (RoB2.0) (41) across the following five core domains: randomization process, deviations from intended interventions, missing outcome data, measurement of the outcome, and selection of the reported result. They will then evaluate the overall risk of bias for the studies. Any disputes will be resolved through consensus or third-party expert adjudication.

### 2.5 Data synthesis and statistical methods

#### 2.5.1 Network plot

We will illustrate the comparative relationships among different insomnia interventions through a network plot. In this visualization, nodes represent individual interventions, with their diameter proportional to the sample size of included studies. Edges indicate direct comparisons between interventions, with thickness reflecting the number of studies available for each comparison.

#### 2.5.2 Pairwise and network meta-analysis

To address heterogeneity arising from the use of different outcome instruments (PSQI and ISI), we will perform separate network meta-analyses for each. The primary analysis will be based on the PSQI, and a secondary analysis on the ISI. Results from these analyses will be presented and interpreted separately. If a study reports both PSQI and ISI, its data will be included in both respective analyses.

The NMA will be conducted within a Bayesian random-effects framework using Markov chain Monte Carlo (MCMC) sampling in R (gemtc package). The model will employ four chains, each with 50,000 iterations after a 25,000-iteration burn-in, thinned by a factor of 10. Prior distributions will be set as N(0, 100²) for treatment effects and Uniform(0, 2) for the between-study standard deviation (τ). Model convergence will be confirmed by ensuring the Gelman-Rubin diagnostic (potential scale reduction factor, R-hat) is below 1.05 for all parameters, supplemented by visual inspection of trace plots. Multi-arm trials will be handled by modeling within-study correlations using a multivariate random-effects model.

For dichotomous outcomes, results will be expressed as odds ratios (ORs) with their 95% credible intervals (CrI). For continuous outcomes, we will use the mean difference (MD) if all studies employ the same instrument; otherwise, the standardized mean difference (SMD) will be calculated. The surface under the cumulative ranking curve (SUCRA) will be used to estimate the probability of each intervention being the most effective. In cases where quantitative synthesis is not feasible, the relevant results will be listed and summarized descriptively.

#### 2.5.3 Assessment of transitivity and consistency

The validity of the network meta-analysis relies on the assumption of transitivity. To evaluate this, we have pre-specified the following potential effect modifiers: baseline insomnia severity, disease duration, presence of mild mood symptoms, and concomitant hypnotic use. We will systematically extract data on these variables across all included studies. The distribution of these variables across treatment comparisons will be summarized in a table; a balanced distribution supports the transitivity assumption, which is essential for integrating direct and indirect evidence (42). If feasible, meta-regression will be employed to assess the impact of continuous modifiers (e.g., baseline severity) on treatment effects. For categorical modifiers (e.g., mood symptoms), subgroup analyses or the node-splitting method will be considered to examine their influence on consistency.

Consistency between direct and indirect evidence will be assessed through both local and global approaches. Local inconsistency will be evaluated with the node-splitting method, which compares differences between direct and indirect evidence, with a P-value < 0.05 indicating statistically significant inconsistency (43). Global consistency will be examined using a design-by-treatment interaction model, which assesses the coherence of the entire network and will be evaluated using the chi-square test (44).

#### 2.5.4 Heterogeneity assessment

Heterogeneity is comprehensively assessed using Cochran’s Q test (*P* < 0.1 suggests heterogeneity), the I² statistic (I² > 50% indicates substantial heterogeneity), and the between-study variance (τ²) (45, 46). If significant heterogeneity is detected, subgroup analysis and meta-regression will be conducted to explore the sources of heterogeneity, and sensitivity analysis (e.g., sequentially excluding studies) will be performed to validate the robustness of the results.

#### 2.5.5 Subgroup analysis and sensitivity analysis

It is important to acknowledge that the included RCTs in this field are often small and single-centered. Consequently, the statistical power of subgroup analyses and meta-regression will be limited. Therefore, all findings from these analyses should be interpreted as exploratory and hypothesis-generating rather than confirmatory.

We plan to group studies based on the following characteristics: (a) type of insomnia diagnosis, (b) study design (control group design, blinding type), (c) treatment duration, and (d) number of treatment sessions.

The robustness of the analysis results will be assessed through sensitivity analysis by excluding (a) studies with high risk of bias, (b) studies with only single-blind design, (c) studies with unclear diagnostic criteria, and (d) studies where any intervention group has n ≤ 10. The results after exclusion will be compared with the main analysis. If there are no substantial changes in the indicators, it will indicate that the overall study results are relatively reliable. If conclusions change, reasons will be transparently reported and discussed.

#### 2.5.6 Synthesis of safety evidence

The primary safety analysis will focus on the dichotomous outcome of participants with ≥1 adverse event. Where data permit, we will perform a network meta-analysis for this endpoint to estimate relative effects and rank interventions by tolerability. An NMA for participants with ≥1 serious adverse event will similarly be attempted to establish a safety ranking. To facilitate a comprehensive clinical assessment, the efficacy ranking (based on the primary outcome) and the tolerability ranking will be presented in parallel within a summary table or figure. Additionally, a structured narrative synthesis detailing the spectrum and severity of specific adverse events will be provided for each intervention.

#### 2.5.7 Publication bias

If the final number of studies included in the analysis is 10 or more, we will assess publication bias. For direct comparisons within closed loops, we will use funnel plots and Egger’s test. However, for the network meta-analysis as a whole, we will primarily use comparison-adjusted funnel plots to evaluate potential publication bias across the network, provided the network is sufficiently connected (47).

#### 2.5.8 Certainty of evidence evaluation

We will assess the confidence in NMA results through the CINeMA online platform (https://cinema.ispm.unibe.ch) (48), a standardized tool developed based on the Grading of Recommendations Assessment, Development, and Evaluation (GRADE) framework (49). This tool evaluates potential biases or limitations in NMA across six domains: (a) within-study bias, (b) reporting bias, (c) indirectness, (d) imprecision, (e) heterogeneity, and (f) incoherence. Each domain is judged at three levels (no concerns, some concerns, or major concerns), ultimately synthesizing an overall confidence rating classified into four grades: high, moderate, low, or very low. In case of disagreement, a third-party expert will make the final judgment.

## 3 Discussion

Insomnia disorder has emerged as a significant global public health challenge, with its high prevalence, comorbid characteristics, and substantial economic costs exerting profound impacts on individual health, healthcare systems, and social resources (50, 51). To address this critical issue, various emerging neurostimulation technologies are being progressively incorporated into chronic insomnia management systems. While existing research has demonstrated that these technologies can improve sleep quality by modulating brain activity, current evidence is primarily limited to head-to-head comparisons of single therapies, lacking evidence-based integrated treatment recommendations. This leaves clinicians facing decision-making dilemmas regarding technology selection and safety assessment.

To bridge the evidence gap in existing clinical guidelines, this study will synthesize data from the latest RCTs. By quantifying differences in core outcome measures before and after interventions, we will systematically evaluate the efficacy and safety profiles of different noninvasive neurostimulation technologies, with evidence quality assessed using the CINeMA. To our knowledge, this will be the first NMA in this field. The findings are anticipated to offer clinicians evidence-based support for clinical decision-making and robust data for updating and refining practice guidelines. Furthermore, this study is expected to accelerate the development and maturation of cutting-edge neuromodulation technologies, promoting their translation from bench to bedside and ultimately providing patients with accessible treatment options and benefits.

Despite our comprehensive search strategy and efforts to contact authors for unpublished data, our study has limitations. The exclusion of conference abstracts and potentially inaccessible unpublished trials may have introduced publication bias, as the literature may be skewed towards larger studies with positive findings. This could lead to an overestimation of the true treatment effects and consequently bias the SUCRA rankings. Therefore, the results and rankings presented in this review should be interpreted with this potential limitation in mind, and they represent the best available evidence primarily from the published literature. Additionally, due to the limited number of available studies, which may prevent in-depth exploration of optimal parameters for each technology, this limitation does not affect our ability to identify the most effective treatment from existing evidence, and the conclusions will still be of great value in filling the gaps in the guidelines.

To promote the translation and application of research findings, we plan to publish this study in a peer-reviewed scientific journal to enhance academic dissemination and guide clinical practice.

## Supplementary material

Additional File 1 PRISMA-P 2015 Checklist.
